# Cost-Effectiveness of Genetic Testing for All Women Diagnosed with Breast Cancer in China

**DOI:** 10.3390/cancers14071839

**Published:** 2022-04-06

**Authors:** Li Sun, Bin Cui, Xia Wei, Zia Sadique, Li Yang, Ranjit Manchanda, Rosa Legood

**Affiliations:** 1 Department of Health Services Research and Policy, London School of Hygiene & Tropical Medicine, London WC1H 9SH, UK; li.sun1@lshtm.ac.uk (L.S.); xia.wei@lshtm.ac.uk (X.W.); zia.sadique@lshtm.ac.uk (Z.S.); r.manchanda@qmul.ac.uk (R.M.); rosa.legood@lshtm.ac.uk (R.L.); 2 Wolfson Institute for Population Health, CRUK Barts Cancer Centre, Queen Mary University of London, London EC1M 6BQ, UK; 3 School of Public Health, Peking University, Beijing 100191, China; cuibin@bjmu.edu.cn; 4 Department of Gynaecological Oncology, Barts Health NHS Trust, Royal London Hospital, London E1 1BB, UK; 5 Department of Gynaecology, All India Institute of Medical Sciences, New Delhi 110029, India

**Keywords:** breast cancer, genetic testing, screening, cost-effectiveness analysis

## Abstract

**Simple Summary:**

Unselected multigene testing at breast cancer (BC) diagnosis has been reported to be cost-effective compared with family history (FH)/clinical-criteria-based testing in high-income countries such as the US and UK. Chinese patients are younger than Caucasian women at diagnosis, tending to have a higher gene mutation prevalence, and the family size and number of female relatives are smaller due to the one-child policy (which has been changed) in China. Therefore, offering genetic testing for BC patients could potentially prevent more cancer cases and deaths in China. However, the health economic evidence for multigene testing at BC diagnosis in China is lacking. The aim of the current study was to evaluate the cost-effectiveness of three genetic testing strategies among BC patients using a microsimulation model at the individual level in China. We found that offering unselected multigene testing to all BC patients in China is highly cost-effective compared with FH/clinical-criteria-based testing or no testing from both the societal and payer perspectives.

**Abstract:**

Unselected multigene testing for all women with breast cancer (BC) identifies more cancer susceptibility gene (CSG) carriers who can benefit from precision prevention compared with family history (FH)/clinical-criteria-based guidelines. Very little CSG testing is undertaken in middle-income countries such as China, and its cost-effectiveness remains unaddressed. We aimed to estimate cost-effectiveness and population impact of multigene testing for all Chinese BC patients. Data from 8085 unselected BC patients recruited to a Peking University Cancer Hospital study were used for microsimulation modeling, comparing three strategies in the Chinese setting: all BC women undergo *BRCA1/BRCA2/PALB2* genetic testing, only BC women fulfilling FH/clinical criteria undergo *BRCA* testing, and no genetic testing. Prophylactic mastectomy and salpingo-oophorectomy would be adopted where appropriate. Societal and payer perspectives with a lifetime horizon along with sensitivity analyses were presented. Incremental cost-effectiveness ratio (ICER): incremental cost per quality-adjusted life-year (QALY) gained is compared to the USD 10,260/QALY (one-times GDP per capita) willingness-to-pay threshold. BC incidence, ovarian cancer (OC) incidence, and related deaths were also estimated. FH/clinical-criteria-based *BRCA* testing was ruled out on the principle of extensive dominance. Compared with no genetic testing, multigene testing for all BC patients had an ICER = USD 4506/QALY (societal perspective) and USD 7266/QALY (payer perspective), well below our threshold. Probabilistic sensitivity analysis showed unselected multigene testing remained cost-effective for 94.2%/86.6% of simulations from the societal and payer perspectives. One year’s unselected multigene testing could prevent 7868 BC/OC cases and 5164 BC/OC deaths in China. Therefore, unselected multigene testing is extremely cost-effective and should be offered to all Chinese women with BC.

## 1. Introduction

Breast cancer (BC) is the most common female cancer worldwide. Knowing a patient’s genetic mutation status can help plan BC management and prognosis. After the diagnosis of unilateral BC, patients carrying an inheritable genetic pathogenic variant (henceforth called ‘path-var’) in a high-risk cancer susceptibility gene (CSG) can undertake surgical prevention to reduce their risk of developing contralateral BC or ovarian cancer (OC). Their unaffected relatives carrying familial path-vars can be identified via cascade testing and benefit from early diagnosis through enhanced BC screening, along with surgical prevention for BC/OC or chemoprevention [1,2]. Additionally, it may influence surgical decision making in some path-var-negative patients who may decide not to undergo contralateral mastectomy [3].

Current international guidelines recommend genetic testing in women with BC fulfilling family history (FH) or clinical criteria. Although technological advancement has led to a rapidly falling cost of genetic testing, to date, the use of CSG testing in routine health care in China has been limited and largely restricted to some large teaching hospitals. While Western health systems are also plagued by limitations to accessibility and uptake of CSG testing [4,5], this is far more restricted and less accessible in China. The prevalence of genetic path-vars and FH among Chinese BC patients are different from those in Western countries. In China, the mean age of BC diagnosis is 45–55 years [6], about 10 years younger than that of Caucasian women [7,8]. Younger BC patients tend to have a higher CSG mutation prevalence [9]; therefore, offering genetic testing for BC patients could potentially prevent more cancer cases and deaths in China. Due to the one-child policy (which has only recently been changed), the family size and number of female relatives are small. Additionally, the sex ratio (100:105) has a skew toward males [10]. This may make paternal inheritance more likely. All this makes the FH-based testing approach potentially more likely to miss path-var carriers in Chinese women. In China, 65.6% of patients with *BRCA1/BRCA2* path-vars do not have a strong FH [11] compared to 15–50% in Western case series [12,13], thus missing huge opportunities for precision prevention using FH-based genetic testing. Offering unselected multigene testing to all BC patients can overcome the limitations of FH/clinical criteria-based testing. *BRCA1/BRCA2* genes have a 69–72% risk of BC [14], while *PALB2* is a more recently established high-penetrance BC gene, associated with a 44–53% BC risk [15,16]. In high-income country health systems such as the US, UK, and Norway, unselected *BRCA1/BRCA2/PALB2* multigene testing at BC diagnosis has been reported to be cost-effective compared with FH/clinical-criteria-based testing [9,17,18] and has led to some calls for policy change [19]. However, the health economic evidence for this approach in middle-income countries such as China is still lacking. For changes in practice to be justifiable and sustainable, it is essential that they are cost-effective for the health system.

In this study, we use data from a large Chinese BC cohort study and from the Chinese Urban Basic Medical Insurance Database, along with modeling to evaluate the cost-effectiveness of CSG testing at BC diagnosis in China. For the first time, we estimated the lifetime health effects, costs, cost-effectiveness, and potential population impact of unselected *BRCA1/BRCA2/PALB2* multigene testing for all BC patients compared to ‘FH/clinical-criteria-based *BRCA* testing’ and with ‘no testing’ in China.

## 2. Methods

We obtained data on FH and CSG prevalence from 8085 unselected BC patients treated at the Breast Centre of Peking University Cancer Hospital from October 2003 to May 2015 [11]. The FH/clinical criteria used for eligibility for testing were based on existing guidelines. Individuals with one or more breast and/or ovarian cancer patients in the first- or second-degree relatives were considered FH positive [11]. For our analysis, we used CSG data for *BRCA1, BRCA2*, and *PALB2*, as these genes have clear clinical applicability for genetic testing [20,21], keeping in mind the principles of analytic validity, clinical validity, clinical utility, and associated ethical/legal/social implications (ACCE framework) [21]. The proportion fulfilling FH/clinical criteria (FH positive) for *BRCA* testing by age group among unselected BC cases was calculated. We obtained population-based BC incidence data from the World Health Organization (GLOBOCAN-2018) [22]. Then, we estimated the total number of FH-positive BC cases based on the number of new invasive BC cases by age group in the Chinese population. Healthcare cost data were accessed from the sampling database of the Chinese Urban Basic Medical Insurance, which represents 50 million of the urban population in China.

### 2.1. Model and Genetic Testing Strategy

**Figure 1:**
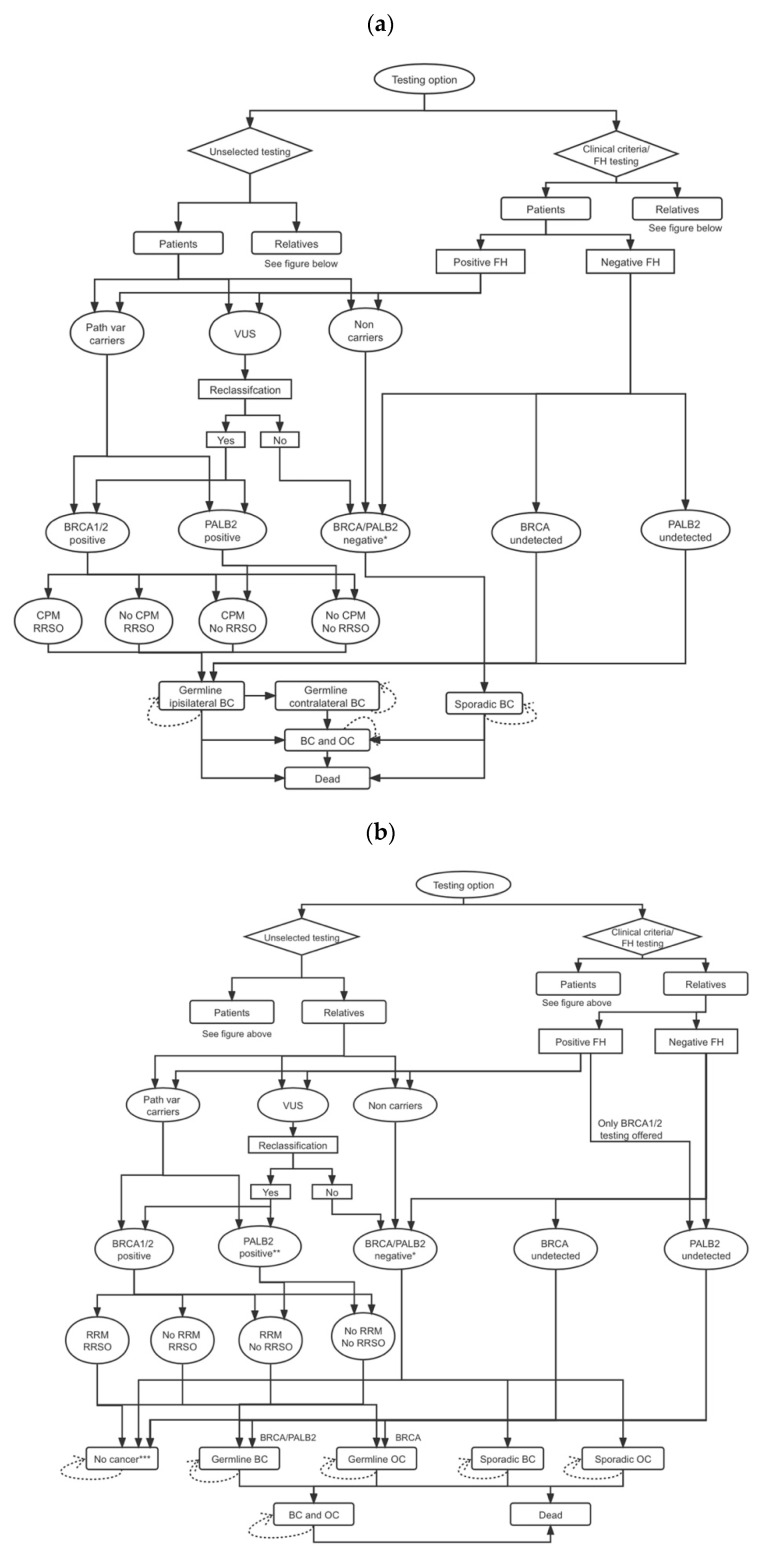
Model structures for unselected and FH/clinical-criteria-based genetic testing for BC patients and relatives: (**a**) for BC patients; (**b**) for relatives of BC patients. Abbreviations: BC, breast cancer; CPM, contralateral prophylactic mastectomy; FH, family history; OC, ovarian cancer; RRM, risk-reducing mastectomy; RRSO, risk-reducing salpingo-oophorectomy; path-var, pathogenic variant. * Includes individuals testing negative for BRCA1/BRCA2/PALB2 mutations and VUS not reclassified as pathologic variants. ** In the model structure for relatives, PALB2-positive individuals are identified only through the unselected testing arm. Relatives in the clinical criteria/FH testing arm only undergo BRCA1/BRCA2 testing. *** Unaffected relatives can progress from no cancer to germline BC (BRCA1/BRCA2/PALB2), germline OC (BRCA1/BRCA2), sporadic BC, or sporadic OC (or remain in that health state).

A microsimulation model at the individual level (see Figure 1; comprehensive explanations are given in Supplementary Materials) was developed (TreeAge-Pro 2018 Williamson, MA, USA) for evaluating the costs and health effects of three genetic testing strategies among BC patients. Strategy A: *BRCA1/BRCA2/PALB2* testing for all BC patients; Strategy B: *BRCA1/BRCA2*-testing for BC patients fulfilling FH/clinical criteria; and Strategy C: no genetic testing. Microsimulation allows for incorporating individual heterogeneity between CSG result and age. Individual patient history can be traced in the model using memories of events (e.g., risk-reducing options) and their impact on future cycles estimated. In Strategy A, all patients with BC undergo genetic testing (unselected testing arm), while in Strategy B, only those fulfilling FH/clinical criteria (FH/clinical-criteria-based testing arm) are offered genetic testing. Initially, first-degree relatives of identified *BRCA1/BRCA2/PALB2* path-var carriers undergo testing, and second-degree relatives are tested if the first-degree relative has a *BRCA1/BRCA2/PALB2* path-var. Our analysis includes the impact of the variant of uncertain significance (VUS), using a VUS rate of 6.4% [23] and a pathogenic/likely pathogenic reclassification rate of 8.7% [24].

Unaffected *BRCA1/BRCA2/PALB2* path-vars can minimize their BC risk through chemoprevention and enhanced BC screening or risk-reducing mastectomy (RRM). *BRCA1/BRCA2* path-vars can minimize OC risk through risk-reducing salpingo-oophorectomy (RRSO). BC path-vars may minimize their contralateral BC risk through CPM. Despite initial studies suggesting that premenopausal RRSO could reduce BC risk [25,26,27], data remain conflicting, with some recent data both contradicting [28] and supporting this [29], leading to uncertainty around this issue. Hence, our scenario analysis evaluates no BC risk reduction. Additionally, our model includes the impact of premenopausal-oophorectomy without hormone replacement therapy (HRT) on excess risk and 3.03% [30,31] absolute increased mortality from coronary heart disease (CHD). In our model (see Supplementary Table S1 for all probabilities), a hypothetical cohort of BC patients and their cancer-free relatives can transition through a range of different health states: no cancer, germline ipsilateral BC, germline contralateral BC, sporadic BC, germline OC, sporadic OC, and both BC and OC. The cancer incidence estimates were determined by adding the pathway probabilities resulting in OC or BC. The decrease in the incidence of BC and OC from testing all BC cases occurring annually in Chinese women was calculated to estimate the overall population impact. A 3% discount rate was used for costs and health outcomes [32].

### 2.2. Probabilities

The different probabilities for various pathways in the model are given in Supplementary Table S1. Data on age-specific incidences of BC and OC in the general population of Chinese women were obtained from the World Health Organization (GLOBOCAN-2018) [22]. Age-specific BC and OC incidence for *BRCA1/BRCA2* path-vars [14], the BC incidence for *PALB2* path-vars [15], and contralateral BC incidence [14] were obtained from the published literature.

### 2.3. Relatives: Number and Age Distribution

The age distribution of new Chinese BC cases was used to calibrate the age distribution of patients in the model [22]. Data from the United Nations World Population Prospects [33] were used to calculate the number of first- and second-degree relatives and their ages relative to BC index cases (see Supplementary Table S2). Age- and gender-specific lifetable data provided estimates for relatives being alive at different ages, and calculation of age distribution and number of relatives undergoing genetic testing.

### 2.4. Costs

Primary data on relevant direct medical costs were obtained from the Urban Basic Medical Insurance Database in China [34]. All costs are reported in 2019 USD, with Chinese RMB values converted to USD using the purchasing power parity (PPP) factor [35] (USD 1 equals RMB 4.21). The analyses were conducted from a societal perspective, as recommended by the Chinese health system [36], as well as a payer perspective recommended by other guidelines [37]. The analysis from the payer’s perspective included the costs of genetic testing costs and BC, OC, and excess CHD treatment. The analysis from the societal perspective included these costs plus costs due to productivity loss, which was associated with disability (temporary and permanent) and premature death. The cost of *BRCA1/BRCA2/PALB2* testing is USD 367, based on the pricing list of genetic testing companies in China. In China, this incorporates/includes the cost of genetic counseling. A comprehensive explanation and summary of costs are given in Supplementary Table S3 and of productivity loss in Supplementary Table S4.

### 2.5. Life-Years

Our study incorporates a lifetime time horizon using lifetime risks and long-term health impact. The life expectancy for women not developing BC/OC was estimated from relevant lifetable data [38]. In the base case, unaffected path-var carriers underwent RRM and RRSO at median ages of 37 and 40 years, respectively [39]. BC and OC survival were modeled using five-year survival data [40] (see Supplementary Figure S1 for details).

### 2.6. Quality-Adjusted Life-Years (QALYs)

As recommended, we use QALYs to measure health outcomes. QALY and utility scores used in the model are explained in Supplementary Figure S1.

### 2.7. Analysis

Simulations were undertaken with the microsimulation model, using the total new Chinese BC cases (367,900) annually and the corresponding number of female relatives (1,252,074) by age. Descriptive validity, technical validity, and face validity were used for internal validation of the model [41]. The difference in lifetime costs between strategies was divided by the difference in lifetime effects (QALYs), to estimate the incremental-cost-effectiveness ratio (ICER). This was compared with the decision maker’s willingness-to-pay (WTP) threshold for a QALY gain of USD 10,262, the one-time GDP per capita in China in 2019 [42].

The principle of extended dominance was applied to compare multiple interventions [43]. The list of three compared interventions, if none strongly dominated, was ordered by effectiveness. Each intervention was compared to the next most effective alternative by calculating the ICER. Extended dominance rules out any intervention that has an ICER that is greater than that of a more effective intervention.

Multiple-scenario analyses were explored: (a) no reduction in BC risk from RRSO; (b) zero HRT compliance; (c) lower surgical prevention (RRM/RRSO) uptake rates; (d) lower RRSO or CPM uptake rates in BC path-vars; (e) lower uptake rates of genetic testing; (f) exclusion of VUS management.

Both one-way (where each variable is varied individually) and probabilistic (where all parameters are varied simultaneously) sensitivity analyses were undertaken to investigate uncertainty of parameters in the model and impact on results. Probabilities and utility scores were varied by their 95% confidence intervals/range where available or by +/−10%, and costs were varied by +/−30%. We used a Gamma distribution for costs, Log-normal distribution for quality of life, and Beta distribution for probability, as recommended [44]. For PSA, we obtained 1000 estimates of incremental costs and effects by sampling from the distributions of each parameter. Cost-effectiveness acceptability curves were drawn for demonstrating the cost-effectiveness probability of different genetic testing strategies for Chinese BC patients at various WTP thresholds.

## 3. Results

The results of the three genetic testing strategies in terms of health effects, costs, and ICERs ordered by effectiveness are given in Table 1. Testing all BC patients reported an ICER of USD 4152/QALY (societal perspective) or USD 6848/QALY (payer perspective) compared to FH/clinical-criteria-based testing, and FH/clinical-criteria-based testing reported an ICER of USD 5416/QALY (societal perspective) or USD 8340/QALY (payer perspective) compared to no testing strategy. FH/clinical-criteria-based testing is ruled out based on the principle of extended dominance, as a greater number of QALYs will be obtained at a lower cost per QALY with the intervention of testing all BC patients.

The base-case analysis results after the dominance principle were applied and FH/clinical-criteria-based testing was ruled out are shown in Table 2. The ICER of testing all BC patients compared to no testing was USD 4506/QALY (societal perspective) and USD 7266/QALY (payer perspective), well below the one-time GDP per capita of USD 10,262 in China. This can lead to an additional 327.7 days’ increased life expectancy for *BRCA1/BRCA2/PALB2* path-var carriers. One year’s unselected genetic testing of all BC patients can prevent an additional 6358 BC cases and 1510 OC cases in China, corresponding to averting 5164 BC/OC deaths in Chinese women over a lifetime horizon (Table 3).

**Figure 2:**
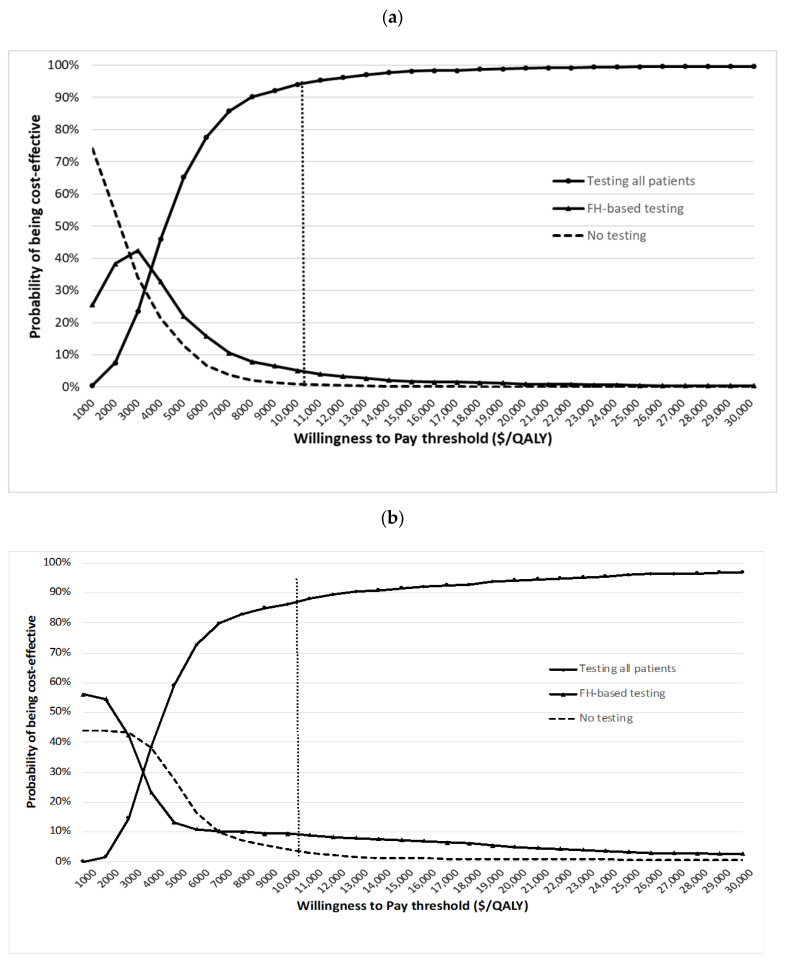
Cost-effectiveness acceptability curves (probabilistic sensitivity analyses): (**a**) cost-effectiveness acceptability curve—societal perspective; (**b**) cost-effectiveness acceptability curve—payer perspective.

One-way sensitivity analysis results show that individual variables such as path-var prevalence, costs, utility scores, and transition probabilities have minimal impact on the cost-effectiveness of unselected testing. A tornado diagram for the top 10 variables is given in Supplementary Figure S1. PSA shows that at the USD 10,262/QALY threshold, unselected genetic testing (compared to FH/clinical-criteria-based or no testing) is cost-effective for 94.2% of simulations from the societal perspective and 86.6% of simulations from the payer perspective (Figure 2). For FH/clinical-criteria-based testing, only 5.1% of simulations are cost-effective from the societal perspective, and 9.4% of simulations are cost-effective from the payer perspective (Figure 2).

A mean of 1.27 unaffected Chinese female relative path-var carriers are identified through cascade testing, which is lower than estimates from other Western populations [9] (see Supplementary Table S2). Unselected multigene testing remains cost-effective across various scenario analyses (see Table 2). These include no BC risk reduction from RRSO (ICER societal = USD 4558/QALY; ICER payer = USD 7308/QALY), zero HRT adherence (ICER societal = USD 4729/QALY; ICER payer = USD 7576/QALY), half RRM uptake rate in unaffected relatives (ICER societal = USD 4777/QALY; ICER payer = USD 7449/QALY), half RRSO uptake rate in unaffected relatives (ICER societal = USD 4735/QALY; ICER payer = USD 7439/QALY), half RRM uptake rate and half RRSO uptake rate in unaffected relatives (ICER societal = USD 5201/QALY; ICER payer = USD 7802/QALY), half CPM uptake rate in patients (ICER societal = USD 5546/QALY; ICER payer = USD 8310/QALY), half RRSO uptake rate in patients (ICER societal = USD 4773/QALY; ICER payer = USD 7588/QALY), lower (70%) genetic testing uptake rate in patients with BC and relatives (ICER societal = USD 5148/QALY; ICER payer = USD 7575/QALY), lower (50%) genetic testing uptake rate in patients with BC and relatives (ICER societal = USD 4739/QALY; ICER payer = USD 6922/QALY), and no VUS management (ICER societal = USD 2848/QALY; ICER payer =USD 5355/QALY) The upper limit of genetic testing costs in China for maintaining cost-effectiveness of unselected genetic testing for all BC patients (given WTP threshold of one-time GDP per capita) is USD 806 per test from the societal perspective and USD 639 per test from the payer perspective.

## 4. Discussion

Our study has for the first time shown that offering unselected multigene testing to all BC patients in China is highly cost-effective compared with FH/clinical-criteria-based testing or no testing from both the societal and payer perspectives. One year’s unselected genetic testing could prevent 7868 BC/OC cases and 5164 BC/OC deaths in China. These results from a middle-income country health system lend further credence to an earlier analysis of the benefits of reductions in BC/OC cases and deaths from cost-effective unselected *BRCA1/BRCA2/PALB2* multigene testing in high-income-country (USA/UK) health systems [9]. This adds to the generalizability of this approach across other (middle-income) health systems. The prevalence of *BRCA* and *PALB2* path-vars appears slightly higher in Chinese BC women [11] than in Caucasian women [45,46]; hence, this may benefit more women with BC and their families in China.

Our study has many strengths. It draws on a large population-based sample of Chinese BC patients, incorporates their earlier age of BC diagnosis, adjusts for the unique (smaller) family size due to the one-child policy in China, includes a detriment for CHD mortality [30], and incorporates costs of counseling and the impact of VUS, HRT use, and osteoprotection. We follow established guidelines for analysis and health economic evaluation [47], present both the societal and payer perspectives with a long-term horizon, use QALYs for health outcomes, integrate utility scores and 3% discounting for costs/outcome, and undertake thorough sensitivity analyses to support the robustness/accuracy of results. Our scenario analyses reconfirm cost-effectiveness without BC risk reduction from RRSO, which is reassuring. RRSO/RRM uptake rates can vary across populations [48], and our scenario analyses demonstrate that unselected genetic testing remains extremely cost-effective, even at 50% lower RRSO/RRM rates. Over the years, genetic testing costs have fallen considerably, but some providers charge higher than our base case. Nevertheless, multigene testing will remain cost-effective until USD 806/test, a value lower than that charged by most providers today.

This study is limited by the lack of direct medical cost data on rural Chinese patients. The sampling database of the Chinese Urban Basic Medical Insurance used in this analysis only covers the urban population. However, differences have been noticed across rural and urban areas in the choice of neoadjuvant chemotherapy and surgical techniques [49], and adherence to adjuvant therapy [50], which could lead to rural–urban differences in the direct medical costs. Although our sensitivity analysis proves that the results are quite robust when the costs are varied up and down by 30%, the impact of cost variations on the overall results could be further explored if more evidence on the treatment costs of rural patients is available. The actual costs of implementation pathways for providing genetic testing in health systems are not well documented and may entail some hidden costs that are being missed. It is thus possible that some administration costs of genetic testing are not included in the analysis. This may lead to an underestimation of costs and can impact the ICER/QALY measure of cost-effectiveness. In addition, in the base-case analysis, we assume all eligible patients undergo genetic testing and all relatives of breast cancer patients with path-vars are successfully cascade tested. However, the uptake rate and the success rate of capturing probands with reclassified VUS results in China are still unknown. This requires careful consideration, and further research is required to reduce uncertainty. Nevertheless, our scenario of a lower uptake rate of genetic testing in patients and relatives demonstrates this approach remains cost-effective. Additionally, health systems can have a range of stakeholders and decision makers at various levels such as the national, regional (example clinical commissioning groups), and local hospital levels (example local budgetary purchases), as well as the individual clinician level [51]. Both perspectives and preferences of these decision makers can vary and impact budgetary implementation, pathway logistics, and final implementation.

We include *PALB2* along with *BRCA1/BRCA2*, as it has clear clinical utility with MRI screening/RRM offered for *PALB2* path-vars. We excluded other high-risk genes such as *STK11/PTEN/p53*, which are pleiotropic syndromic and very rare or associated with only a small subset of BC (lobular) and lack reliable risk estimates corrected for ascertainment bias [20]. *ATM* and *CHEK2* have lower risks (RR= ~1.5–2.0), and RRM is not routinely offered, with many clinicians opining they lie below the threshold for clinical intervention [20]. There have been concerns around clinician interpretation and inappropriate management such as RRM for VUS and moderate-risk genes that do not reach the threshold for clinical intervention. As testing for *ATM* and *CHEK2* is not currently routinely part of clinical practice, we did not include these in our analysis. However, recent modeling data highlight the possible benefits of breast screening with MRI in this population [52]. It is probable that testing for these CSGs may be introduced in the future for no additional cost, which could further improve cost-effectiveness. Additionally, future potential includes options of further risk-adapted management incorporating other genetic and non-genetic risk factors to improve the precision of the risk estimate, such as polygenic-risk-score and other epidemiological, family history, reproductive/hormonal factors, and mammographic density. *PALB2* was recently reported to be a moderate OC-risk gene (OC risk = 5%) [16]. We have been conservative and not included the benefit of RRSO and OC risk reduction in *PALB2* path-vars, as practice has not yet changed across health systems. If included, it would further improve cost-effectiveness.

Current practice is afflicted by massive underutilization of genetic testing resulting in missed opportunities for BC/OC prevention. Using age or clinically based criteria is a restrictive practice, which will miss high-risk women. An unselected BC testing approach will maximize the number of BC patients diagnosed with path-vars. It can provide a huge stimulus for early diagnosis/prevention in unaffected family members besides clinical benefits for the BC patient. These women can undergo bilateral mastectomy at initial BC surgery instead of breast conservation. It can minimize contralateral BC risk, possibly avoid adjuvant radiotherapy, and provide better alternatives for breast reconstruction [53], while having potential therapeutic implications such as PARP inhibitors. They are at increased risk of OC and can undergo RRSO following BC treatment. The American Society of Breast Surgeons [19] and others [9,12] have called for adopting an unselected genetic testing strategy for all at BC diagnosis, but the NCCN guidelines and the American College of Medical Genetics and Genomics (ACMG) have not yet supported this [54]. Hence, this approach has not yet been adopted in clinical practice. The *BRCA-DIRECT* study is evaluating the implementation of unselected genetic testing at BC diagnosis in the UK.

Despite its cost-effectiveness and clinical benefit, a number of challenges need addressing in the implementation of a policy supporting unselected multigene testing for all BC patients across China. Financial difficulty from out-of-pocket costs can be a barrier for Chinese BC patients, as genetic testing is currently not covered by the National Basic Health Insurance in China. The Chinese health system needs to consider incorporating cost-effective genetic testing interventions with significant patient benefit such as this into their National Health Insurance plan. Expansion in local laboratory infrastructure is needed to improve access and manage throughput, as genetic testing is currently mainly performed through laboratories at major hospitals affiliated with top-ranked universities [55]. Given the volume of BC cases diagnosed annually, newer implementation approaches such as ‘mainstreaming’ genetic counseling and testing, which have been successfully implemented across OC treatment pathways [56], will be needed for the successful large-scale implementation of testing at BC diagnosis too. Given the increasing applicability of genetics to cancer care and prevention, most cancer clinicians will need to be trained to improve their understanding of genetics and counsel patients. Implementation will need to be accompanied by a process of education for clinicians and health professionals involved in the BC patient care pathway. There is also a need to expand resources/infrastructure and clinical efforts for downstream management pathways (including screening and prevention) of unaffected high-risk women identified through genetic testing.

## 5. Conclusions

In summary, our analysis demonstrates that an unselected *BRCA1/BRCA2/PALB2* multigene testing strategy is highly cost-effective in China, can prevent thousands of BC/OC cases/deaths, and provides a basis for policy change to implement this. For China, it can be more advantageous to move straight to unselected genetic testing for all BC patients from the current predominant situation of ‘no testing’ rather than first adopting FH/clinical-criteria-based testing currently practiced in most countries.

## Figures and Tables

**Table 1:** Lifetime discounted costs, effects and ICER before dominance principle applied.

Interventions	Health Effects		Costs (USD)			ICER (Cost/QALY)	
	LYGs	QALYs	Payer	Societal		Payer	Societal
Testing all BC patients	14.164	13.483	4686	6808	Testing all BC patients vs. testing based on FH/clinical criteria	6848	4152
Testing based on FH/clinical criteria	14.149	13.470	4596	6753	Testing all BC patients vs. no testing	8340	5416
No testing	14.144	13.465	4554	6726	-	-	-

Abbreviations: BC, breast cancer; FH, family history; ICER, incremental cost-effectiveness ratio; LYG, life-years gained; QALY, quality-adjusted life-year.

**Table 2:** Lifetime discounted costs and effects per woman and ICER after genetic testing for all patients with BC ^a^.

Testing all BC Patients				No Testing				ICER			
Health Effects		Costs (USD)		Health Effects		Costs (USD)		Cost/LYG		Cost/QALY	
LYGs	QALYs	Payer	Societal	LYGs	QALYs	Payer	Societal	Payer	Societal	Payer	Societal
Baseline											
14.164	13.483	4686	6808	14.144	13.465	4554	6726	6509	4037	7266	4506
Scenario: No reduction in BC risk from RRSO ^b^											
14.164	13.483	4686	6808	14.144	13.465	4554	6726	6508	4060	7308	4558
Scenario: No HRT Adherence ^c^											
14.163	13.483	4687	6809	14.144	13.465	4554	6726	6730	4201	7576	4729
Scenario: Half RRM uptake in unaffected relatives ^d^											
14.164	13.483	4687	6811	14.144	13.465	4554	6726	6546	4198	7449	4777
Scenario: Half RRSO uptake in unaffected relatives ^e^											
14.163	13.482	4682	6807	14.144	13.465	4554	6726	6425	4090	7439	4735
Scenario: Half RRM and half RRSO uptake in unaffected relatives ^f^											
14.164	13.482	4685	6813	14.144	13.465	4554	6726	6514	4342	7802	5201
Scenario: Half CPM uptake in patients ^g^											
14.160	13.481	4683	6812	14.144	13.465	4554	6726	7857	5243	8310	5546
Scenario: Half RRSO uptake in patients ^h^											
14.160	13.481	4672	6800	14.144	13.465	4554	6726	7014	4412	7588	4773
Scenario: Lower uptake rate of genetic testing in patients and relatives ^i (70%)^											
14.158	13.477	4644	6787	14.144	13.465	4554	6726	6229	4233	7575	5148
Scenario: Lower uptake rate of genetic testing in patients and relatives ^i (50%)^											
14.153	13.473	4607	6762	14.144	13.465	4554	6726	5449	3731	6922	4739
Scenario: No VUS management ^j^											
14.162	13.479	4629	6766	14.144	13.465	4554	6726	3943	2097	5355	2848

Abbreviations: BC, breast cancer; HRT, hormone replacement therapy; ICER, incremental cost-effectiveness ratio, LYG, life-years gained; QALY, quality-adjusted life-year; RRM, risk-reducing mastectomy; RRSO, risk-reducing salpingo-oophorectomy. ^a^ Discounted at 3%; ^b^ Probability P15 = 1 (Table S1 in the Supplement); ^c^ Probability P21 = 0 (Table S1 in the Supplement); ^d^ Probability P9 = 0.235 (Table S1 in the Supplement); ^e^ Probability P11 = 0.275 (Table S1 in the Supplement); ^e^ Probability P9 = 0.235 and Probability P11 = 0.275 (Table S1 in the Supplement); ^g^ Probability P10 = 0.270 (Table S1 in the Supplement); ^h^ Probability P12 = 0.284 (Table S1 in the Supplement); ^i (70%)^ Indicates a genetic testing uptake rate of 70%; ^i (50%)^ Indicates a genetic testing uptake rate of 50%; ^j^ Indicates no VUS management.

**Table 3:** Population effect of genetic testing for patients with BC.

IMPACT	Testing All BC Patients		Testing Based on Family History		No Testing		Difference (Testing All vs. No Testing)		
	Patients	Relatives	Patients	Relatives	Patients	Relatives	Patients	Relatives	Total
Germline BC cases	2075 ^a^	7658	3806 ^a^	10,493	4515 ^a^	11,576	2440	3918	6358
Germline OC cases	737	2144	1263	2640	1487	2904	750	760	1510
Germline BC/OC deaths	4873	3679	7237	4968	8247	5469	3374	1790	5164

^a^ Indicates contralateral breast cancer cases in patients with unilateral breast cancer (breast cancer in one breast). This table depicts the additional BC and OC cases and deaths prevented by an unselected BC testing strategy.

## Data Availability

Data supporting the findings of this study are available in the supplementary material of this article.

## Supplementary Materials

The following supporting information can be downloaded at: https://www.mdpi.com/article/10.3390/cancers14071839/s1, Figure S1: Tornado diagram – One-way Sensitivity Analysis. (a) One-way sensitivity analysis from societal perspective; (b) One-way sensitivity analysis from payer perspective, Table S1: Probabilities of different pathways in the model and explanations, Table S2: Generating cohort of relatives, Table S3: Summary of medical costs used in the model (2019 prices) and explanation, Table S4: Descriptive statistics for productivity loss in breast and ovarian cancer patients.
