# Supplementary material for: Cost-Effectiveness of Genetic Testing for All Women Diagnosed with Breast Cancer in China
Source: Cancers (Basel). 2022 Apr 6;14(7):1839. doi: 10.3390/cancers14071839 (PMC8997428; doi:10.3390/cancers14071839)
Supplement: Supplementary file 1 [file cancers-14-01839-s001.zip › cancers-1617113-Supplementary.pdf]

Supplementary Material: Cost-Effectiveness of Genetic Testing for All Women Diagnosed with Breast Cancer in China

Li Sun, Bin Cui, Xia Wei, Zia Sadique, Li Yang, Ranjit Manchanda and Rosa Legood

1. Microsimulation Model Structure for BC Patients and Relatives.

1.1. Patients in Unselected Testing Arm

In the unselected testing arm, all breast cancer (BC) patients are offered genetic testing and get classified as path var carriers, VUS, or non-carriers. A proportion of VUS patients (8.7%) get subsequently reclassified as path var carriers.

BRCA1/*BRCA2* BC path var carriers are offered contralateral prophylactic mastectomy (CPM) and risk-reducing salpingo-oophorectomy (RRSO). *PALB2* BC path var carriers can undergo CPM. Depending on the probability of patients undertaking a CPM and/or RRSO they may progress to either germline contralateral BC or both BC and OC. Additionally they have a probability of dying from germline BC. Patients who do not progress or die stay in the state of germline ipsilateral BC and undertake the next cycle.

BRCA1/*BRCA2*/*PALB2* negative patients have sporadic BC. Age-dependent probabilities allow them to develop sporadic OC and progress to the health state of 'BC and OC'. They have a probability of dying from sporadic BC. Women who do not progress to BC and OC or die would stay in the health state of sporadic BC to undertake the next cycle.

1.2. Patients in Clinical Criteria/FH Testing Arm

In the clinical criteria/FH testing arm, patients with positive FH undergo genetic-testing and get classified as path var carriers, VUS, or non-carriers. A proportion of VUS patients (8.7%) get subsequently get reclassified as path var carriers.

Patients with negative FH do not undergo genetic testing. They can be undetected *BRCA1*/*BRCA2* path var carriers, undetected *PALB2* path var carriers, or *BRCA1*/*BRCA2*/*PALB2* negative non-carriers.

Options of CPM/RRSO and disease progression for identified *BRCA1*/*BRCA2*/*PALB2* BC path var carriers and disease progression for *BRCA1*/*BRCA2*/*PALB2* negative BC patients, is the same as those in the unselected testing arm and are described above.

Undetected *BRCA1*/*BRCA2* path var carriers are not offered CPM or RRSO, and undetected *PALB2* path var carriers are not offered CPM. Depending on their baseline risk they progress to either germline contralateral BC or both BC and OC. Also they have a probability of dying from germline BC. Patients who do not progress or die would stay in the state of germline ipsilateral BC and undertake the next cycle.

1.3. Relatives in the Unselected Testing Arm

In the unselected testing arm, relatives of BC path var carriers are offered *BRCA1*/*BRCA2*/*PALB2* genetic testing and classified as path var carriers or non-carriers. Relatives of BC patients with *BRCA1*/*BRCA2*/*PALB2* VUS (8.7%) who get reclassified as path var carriers also get offered predictive testing.

Relatives identified with *BRCA1*/*BRCA2* path vars are offered options of risk-reducing mastectomy (RRM) and RRSO, and those with *PALB2* path vars are offered RRM. Unaffected relatives can also opt for chemoprevention for BC. Depending on the probability of path var carriers undertaking an RRM and/or RRSO (+/− chemoprevention) they progress to either germline BC (*BRCA1*/*BRCA2*/*PALB2*) or germline OC (*BRCA1*/*BRCA2*), or stay in a health state of no cancer. They have a probability of dying from the background all-cause mortality.

BRCA1/*BRCA2*/*PALB2* negative women progress to sporadic BC or sporadic OC, or stay in the health state of no cancer. They have a probability of dying from the background all-cause mortality.

1.4. Relatives in the Clinical-Criteria/FH Testing Arm

In the clinical-criteria/FH testing arm, relatives of identified *BRCA1*/*BRCA2* path var patients undergo predictive *BRCA1*/*BRCA2* genetic testing. They are classified as path var carriers, or non-carriers. Relatives of BC patients with VUS who get reclassified as path var carriers also undergo predictive *BRCA1*/*BRCA2* testing.

PALB2 path var carriers cannot be detected with only FH based *BRCA1*/*BRCA2* genetic testing being offered. Relatives of patients with negative FH may be undetected *BRCA1*/*BRCA2* path var carriers, undetected *PALB2* path var carriers, or *BRCA1*/*BRCA2*/*PALB2* negative.

The options of RRM and RRSO for identified carriers are the same as in the unselected testing arm. For identified *BRCA1*/*BRCA2*/*PALB2* path var carriers and non-carries (*BRCA1*/*BRCA2*/*PALB2* negative), the disease progression is the same as relatives in the unselected testing arm.

Undetected *BRCA1*/*BRCA2* path var carriers do not undergo RRM or RRSO, and undetected *PALB2* path var carriers do not undergo RRM. Depending on their baseline risk they progress to either germline BC or germline OC, or stay in 'no cancer' health state. They also have a probability of dying from the background all-cause mortality.

2. Probabilities of Different Pathways in the Model and Explanations

Table S1. Probabilities of different pathways in the model and explanations.

Probability	Value	(95% CI) (Range)	Description	Source
P1	0.053	(0.049,0.058)	BRCA1/BRCA2 mutation prevalence in unselected breast cancer patients	[1]
P2	0.007	(0.005, 0.009)	PALB2 mutation prevalence in unselected breast cancer patients	[1]
P3	0.100	(0.093,0.106)	Probability of having a positive FH among unselected patients	[1]
P4	0.181	(0.155,0.210)	BRCA1/BRCA2 mutation prevalence in FH-positive patients	[1]
P5	0.024	(0.014,0.037)	PALB2 mutation prevalence in FH-positive patients	[2]
P6	0.0453	(0.0350,0.0585)	BRCA1/BRCA2 VUS prevalence in breast cancer patients	[3]
P7	0.0186	(0.0130,0.0264)	PALB2 VUS prevalence in breast cancer patients	[3]
P8	0.0869	(0.0755,0.0999)	Reclassification rate of VUS	[4]
P9	0.47	(0.34,0.56)	Uptake of RRM in unaffected mutation carriers	[5]
P10	0.539	(0.442,0.636)	Uptake of CPM in carriers with breast cancer	[6]
P11	0.55	(0.45,0.64)	Uptake of RRSO in unaffected carriers	[7]
P12	0.567	(0.506,0.629)	Uptake of RRSO in carriers with breast cancer	[8]
P13	0.911	(0.62,0.98)	Reduction in breast cancer risk from RRM without RRSO in unaffected mutation carriers	[9]
P14	0.95	(0.78,0.99)	Reduction in breast cancer risk from RRM with RRSO in unaffected mutation carriers	[9]
P15	0.49	(0.37,0.65)	HR for breast cancer from RRSO alone in unaffected mutation carriers	[10]

P16	0.18	(0.07,0.45)	HR for contralateral breast cancer risk from CPM after breast cancer diagnosis	[6]
P17	0.35	(0.20,0.61)	HR for contralateral breast cancer risk from RRSO after breast cancer diagnosis	[11]
P18	0.96	(0.8,0.96)	Reduction in ovarian cancer risk from RRSO	[10,12]
P19	0.46	(0.27,0.79)	HR for breast cancer survival from RRSO	[13]
P20	0.37	(0.17,0.80)	HR for breast cancer survival from CPM	[6]
P21	0.8	(0.76,0.83)	Compliance of HRT	[14]
P22	0.71	(0.60,0.83)	HR of breast cancer risk from chemoprevention	[15]
P23	0.163	(0.136,0.19)	Uptake of breast cancer chemoprevention	[16]
P24	0.0072	(0.0068,0.0076)	Annual excess risk of developing CHD after RRSO	[17]
P25	0.0303	(0.011,0.043)	Cumulative mortality from CHD after RRSO without HRT	[17]

95%CI - 95% confidence interval, CHD - coronary heart disease, CPM - contralateral prophylactic mastectomy, FH - family history, HR - Hazard Ratio, HRT - hormone replacement therapy, RRSO - risk-reducing salpingo-oophorectomy, RRM - risk-reducing mastectomy, VUS - variant of uncertain significance.

Explanations:

P1–P2: The probabilities of carrying a *BRCA1/BRCA2* (P1) or *PALB2* (P2) Pathogenic variant (path var) in unselected breast cancer patients are taken from a population based Chinese study (Sun et al 2017) of 8,085 consecutive unselected breast cancer patients[1].

P3: The proportion of having a positive family history is obtained from the Chinese study by Sun et al 2017.[1] 805 patients among 8,085 unselected breast cancer cases have a positive family history.

P4: The overall *BRCA1/BRCA2* mutation prevalence among FH positive breast cancer patients is taken from Sun et al 2017[1].

P5: The probability of carrying a *PALB2* mutation in breast cancer patients with a positive FH is calculated based on Sun et al 2017[1].

P6–P7: We obtained the *BRCA1/BRA2/PALB2* VUS prevalence from a systematic review and meta-analysis by van Marcke et al 2018 including 1,870 breast cancer patients[3]. VUS rate to be 1.23% for *BRCA1*, 3.29% for *BRCA2* and 1.86% for *PALB2* in high-risk breast cancer patients.[3] This gives a total VUS rate of 6.4%[3].

P8: The reclassification rate of VUS is taken from Mersch et al 2018 [4]. 8.69% of VUS (178 of 2048) were upgraded to pathogenic or likely pathogenic variants.

P9: The probability that unaffected carriers will undergo RRM is taken from an analysis of UK *BRCA1/2* carriers by Evans et al 2009 [5]. A composite uptake rate for *BRCA1* (60% RRM rate) and *BRCA2* (43% RRM rate) carriers weighted for the relative prevalence of *BRCA1 and BRCA2* mutations was computed [5].

P10: The uptake of CPM in *BRCA1/BRCA2* women diagnosed with unilateral breast cancer is obtained from a cohort study by Evans et al 2013 in the UK [6].

P11: The uptake of RRSO in unaffected *BRCA1/BRCA2* carriers is taken from a study among high-risk UK women [7].

P12: The uptake of RRSO in women with *BRCA1/BRCA2* breast cancer is taken from Kauff et al 2008[8].

P13: The reduction in breast cancer risk from RRM in *BRCA1/BRCA2* mutation carriers not undergoing RRSO is taken from the PROSE study data by Rebbeck et al 2004 [9].

P14: The reduction in breast cancer risk in *BRCA1/BRCA2* mutation carriers undergoing RRM and RRSO is taken from the PROSE study data by Rebbeck et al 2004 [9].

P15: The Hazard Ratio for breast cancer in pre-menopausal unaffected *BRCA1/BRCA2* women undergoing RRSO alone is taken from a meta-analysis by Rebbeck et al 2009 [10].

P16: The Hazard Ratio for contralateral breast cancer risk from CPM in women with *BRCA1*/*BRCA2*-associated breast cancer is obtained from Evans 2013 [6].

P17: The Hazard Ratio for contralateral breast cancer risk from RRSO in *BRCA1*/*BRCA2* mutation carriers after breast cancer diagnosis is obtained from a UK study by Basu 2015 [11], using data from the regional genetics service and the family history clinic at the Genesis Breast Cancer Prevention Centre in Manchester.

P18: The reduction in ovarian cancer risk obtained from RRSO is taken from previous studies which report a 4% residual-risk of primary peritoneal cancer following RRSO [12].

P19: The Hazard Ratio for breast cancer survival from RRSO is obtained from Metcalfe 2015 [13].

P20: The Hazard Ratio for breast cancer survival from CPM is obtained from Evans 2013 [6].

P21: HRT compliance rate is obtained from a UK cohort (Read et al, 2010) [14].

P22: The Hazard Ratio for breast cancer risk from chemoprevention in high-risk women is obtained from the extended long-term follow-up of the IBIS-I breast cancer prevention trial (Cuzick et al 2015) [15].

P23: The uptake of breast cancer chemoprevention is obtained from a recent meta-analysis by Smith et al 2016 [16].

P24: Excess risk of CHD after RRSO is estimated using data from Parker 2013 [17]. The absolute excess CHD incidence is obtained by subtracting CHD incidence in women undergoing RRSO from those not.

P25: The risk of CHD mortality is obtained from the Nurses Health Study (Parker et al 2013) [17]. Death from CHD is reported in 1 in 33 pre-menopausal women undergoing RRSO and not taking HRT [17].

3. Generating Cohort of Relatives

Table S2. Generating cohort of relatives.

**First-degree relatives**	**Mother**	**Father**	**Siblings**	**Children**
Average number	1	1	0.69	1.69
Age relative to index case	28	30	0	−28
Sex, probability female	100%	0%	48.68%	48.68%
Probability mutation	50%	50%	50%	50%
Second-degree relatives	Grandparents	Uncle/aunts	Nieces/nephews	Grandchildren
Average number	4	1.38	1.17	2.86
Age relative to first-degree relatives	28	0	−28	−28
Sex, probability female	50%	48.68%	48.68%	48.68%
Probability mutation	25%	25%	25%	25%
Reference	United Nations World Population Prospects [18]			

The average number of first or second-degree relatives, ages relative to index cases, and the probability of being female are derived from the United Nations World Population Prospects [18]. The number of breast cancer cases by age group is reported by the World Health Organisation (GLOBOCAN-2018) [19]. Based on the average number of relatives and the age relative to the index cases (see table above), we calculated the number of first-/second-degree relatives at different ages. Then we used the lifetables based on age and gender [20] to obtain the probability of being alive for relatives at different ages and to calculate the number of relatives that need to be tested. The probability of carrying a path-var/mutation in a first-degree relative of a known mutation carrier (following predictive testing) is 50%. The probability of carrying a path-var/mutation in a second-degree

relative of a known mutation carrier (following predictive testing) is 25%. The number of unaffected female relative path var carriers identified through cascade testing is calculated to be 1.27 per index path var carrier with BC in China. Male first-degree relatives were tested to inform the need to test second-degree relatives but they were not followed in the model. Long-term outcomes-&-costs were only modelled for females.

4. Summary of Medical Costs Used in the Model (2019 Prices) and Explanation

Table S3. Summary of medical costs used in the model (2019 prices) and explanation.

Item	RMB	USD
Cost of genetic testing	2534	367
Cost of RRSO (and HRT and osteoporosis prevention)	26,881	3896
Cost of ovarian cancer diagnosis and initial treatment	14,907	2161
Yearly cost of ovarian cancer treatment and follow-up: years 1–2	55,651	8065
Yearly cost of ovarian cancer treatment and follow-up: years 3–5	55,109	7987
Terminal care cost with ovarian cancer	11,545	1673
Cost of breast cancer screening general	1223	177
Cost of breast cancer screening mutation carriers	12,767	1850
Cost of RRM (and reconstruction and complications)	5414	785
Cost of CPM (and reconstruction and complications)	4045	586
Cost of chemoprevention	536	78
Cost of breast cancer diagnosis and initial treatment (Sporadic, PALB2)	82,148	11,905
Cost of breast cancer diagnosis and initial treatment (BRCA1/BRCA2)	73,368	10,634
Yearly cost of breast cancer follow-up and adjuvant treatment: years 1-5 (Sporadic)	13,275	1925
Yearly cost of breast cancer follow-up and adjuvant treatment: years 1-5 (BRCA1/BRCA2)	11,464	1662
Yearly cost of breast cancer follow-up and adjuvant treatment: years 1-5 (PALB2)	13,496	1955
Terminal care cost with breast cancer	11,545	1673
Cost of fatal CHD	12,881	1867
Cost of excess CHD	13,025	1888

CPM – contralateral prophylactic mastectomy, HRT – hormone replacement therapy, RRSO – risk-reducing salpingo-oophorectomy, RRM – risk-reducing mastectomy. Model costs are estimated at 2016 prices.

Explanations:

We collected primary data on relevant direct medical costs from the Urban Basic Medical Insurance Database in China[21]. All costs are adjusted for 2019 price index. We convert Chinese RMB values to 2019 USD dollars using purchasing-power-parity (PPP) factor[22] and consumer price index (CPI). Costs of breast cancer (BC), ovarian cancer (OC) and excess coronary heart disease (CHD) are included. In line with NICE recommendations, future healthcare costs not associated with BC, OC, or CHD were not considered[23].

4.1. Cost of Genetic Testing/Counselling

The cost of *BRCA1*/*BRCA2*/*PALB2* testing is $367 based on the pricing list of genetic testing companies in China.

4.2. RRSO Costs

The RRSO cost and HRT cost are obtained from the Urban Basic Medical Insurance Database. Costs include the cost of three follow up DEXA scans for monitoring bone health and calcium and vitamin-D3 for additional osteo-protection. Costs assume HRT is given from average age of RRSO to the average age of menopause (51 years). These costs are calculated for the 80% assumed to be compliant with HRT.

4.3. RRM and CPM Costs

The RRM and CPM costs are obtained from the Urban Basic Medical Insurance Database. Reconstruction rates of around 91% have been reported after RRM [24]. For RRM and reconstruction we assume a 26.2% minor complication rate and 5.6% major complication rate [25], additional costs for which have been included for both minor and major complications [25]. Reconstruction rate after contralateral prophylactic mastectomy (CPM) is 90% [26]. Complication rates for contralateral mastectomy are higher than unilateral mastectomy and the major complication rate with reconstruction is higher than without reconstruction. The complication rate for contralateral mastectomy without reconstruction is 42.9% (40.9% minor and 2% major)[26] and the complication rate for contralateral mastectomy and reconstruction is 41.6% (27.7% minor and 13.9% major) [26].

4.4. Costs of Ovarian Cancer Treatment

We assume that the costs of ovarian cancer diagnosis include a pelvic examination, ultrasound scan, CA125 test, CT scan, percutaneous biopsy, and peritoneal cytology. The costs of ovarian cancer treatment include the reference cost for a lower and upper genital tract very complex major procedure and administration of chemotherapy based on 6 cycles of carboplatin and paclitaxel treatment. It is assumed that in the first and second years treated survivors would have a further three consultant visits, a CT scan and four CA125 tests each year. In the third to fifth years post-surgery, it is assumed that survivors would have two consultant visits and two CA125 tests. Costs for ovarian cancer diagnosis, treatment, and recurrence are derived from the Urban Basic Medical Insurance Database. In line with NICE recommendations future healthcare costs not associated with ovarian cancer are not considered [23].

4.5. Cost of Breast Cancer Screening

For non-carriers, we assume routine biennial mammography between 45-69 years for non-carriers according to the breast cancer screening guideline for Chinese women [27] (13 mammograms on average). For *BRCA1*/*BRCA2*/*PALB2* mutation carriers, we assume annual mammogram and MRI starting at 30 years, and annual mammography only from 50 years [27].

4.6. Cost of Chemoprevention

BRCA1/*BRCA2* mutation carriers are offered Tamoxifen (premenopausal) or Raloxifene (postmenopausal) for 5 years [28,29] to reduce breast cancer risk. The drug costs are obtained from the Urban Basic Medical Insurance Database. 16.3% uptake is assumed for chemoprevention [16].

4.7. Costs of Breast Cancer Treatment

In the general population, 10% breast cancer is non-invasive DCIS and 90% is invasive. 96.7% of invasive breast cancer is early and locally advanced (stage 1–3), and 3.3% of invasive breast cancer is advanced breast cancer (stage 4) [30]. In *BRCA1/2* carriers, 20% of cancers are DCIS and 80% invasive [31,32].

70% of invasive breast cancers are ER-positive [33,34], among which 49% are premenopausal. 15% of early/locally advanced breast cancers and 25% of advanced breast cancers are HER2-positive. 27% *BRCA1* and 67% *BRCA2* breast cancers are ER-positive; 5% *BRCA1* and 14% *BRCA2* breast cancers are HER2-positive [35–40]. All costs are adjusted for *BRCA1*/*BRCA2* breast cancers for differences in stage at presentation, the proportion of being non-invasive, and the proportion of being ER-positive or HER2-positive.

Diagnosis costs: Diagnosis in the breast clinic is made by triple assessment (clinical assessment, mammography, and ultrasound imaging with core biopsy and/or fine needle aspiration cytology) [33]. For all patients presented with suspected advanced breast cancer, MRI should be offered to assess for bone metastases [34].

Sentinel lymph node biopsy (SLNB) costs: SLNB is used for staging axilla for early invasive breast cancer and no evidence of lymph node involvement on ultrasound or a negative ultrasound-guided needle biopsy (73% of early and locally advanced invasive cancers).

Pre-treatment axilla ultrasound costs: Pre-treatment ultrasound evaluation of the axilla should be performed for all patients being investigated for early invasive breast cancer and, if morphologically abnormal lymph nodes are identified, ultrasound-guided needle sampling should be offered [33]. The commissioning cost of pre-treatment ultrasound evaluation of the breast and axilla is the same as that of the breast only [41]. The costing model considers the cost of ultrasound-guided needle sampling only.

Axillary lymph node dissection (ALND) costs: ALNB is undertaken for lymph node positive cancers (~31% early and locally advanced invasive cancers [33,41,42]; 30% node positive for BRCA1/2 breast cancer)[31,35–37,43]. Cost of ALND is assumed to be 25% of the cost of breast surgery as per NICE guideline development group recommendation [41].

Breast surgery costs include costs of breast conserving surgery (assumed for all non-invasive cancers, and 75% of early/locally advanced invasive cancers) and costs of mastectomy (for 25% early/locally advanced and all advanced cancers). Reconstruction rate following mastectomy is assumed to be 34% [44]. The complication rate following mastectomy alone is 21.5% (19.5% minor and 2% major) [26] and complication rate following mastectomy and reconstruction is 28.6% (24.5% minor & 4.1% major)[26]. Costs are obtained from the Urban Basic Medical Insurance Database.

Chemotherapy and radiotherapy costs: Invasive breast cancers who are not at low risk [42,45,46] receive adjuvant treatment. Costs include radiotherapy costs for 60% of early invasive/locally advanced, radiotherapy and chemotherapy costs for 40% early invasive/locally advanced, and chemotherapy for all advanced cancers. Chemotherapy costs based on polychemotherapy [43], include administration costs, costs of 1st and 2nd line therapy and toxicity from NICE guidelines [34,41].

Endocrine therapy costs: ER-positive invasive breast cancers receive Tamoxifen 20mg/day (premenopausal) or Anastrazole 1mg/day (postmenopausal). 70% of invasive breast cancers are ER-positive [33,34], among which 49% are premenopausal. We assume the length of endocrine therapy is 5 years. The drug costs are obtained from the Urban Basic Medical Insurance Database.

Target therapy costs: HER2-positive breast cancer patients can be given at 3-week intervals for 1 year or until disease recurrence. Breast cancer patients with positive HER2 are eligible for treatment with trastuzumab [33,34]. 10% of the eligible patients are intolerant of trastuzumab. Among women suitable for this treatment, 80% receive trastuzumab [41]. HER2 testing costs are obtained from a Chinese tertiary hospital and included for all invasive breast cancers. The trastuzumab cost per patient is obtained from the Urban Basic Medical Insurance Database.

Follow up costs: Breast cancer patients are offered mammographic surveillance and clinical follow-up. We assume patients are followed up every four months in the first two years, and every six months from the third to the fifth year.

Bisphosphonate costs: Bisphosphonates is considered to be offered to patients newly diagnosed with bone metastases, to prevent skeletal-related events and reduce pain [34]. 74% patients with advanced breast cancer will develop bone metastases and 65% patients with bone metastases are offered bisphosphonates[41,47]. Bisphosphonates that are currently offered include oral sodium clodronate, ibandronic acid, zoledronic acid, and pamidronate. The proportions of patients receiving the four drugs are 20%, 30%, 25%, and 25% respectively. We assume the average length of bisphosphonates treatment is 2.7 years, which is the life expectancy of advanced breast cancers based on one-year survival rate (63.2%) [48]. The bisphosphonate costs are obtained from the Urban Basic Medical Insurance Database.

Recurrence costs: For non-invasive breast cancers, the non-invasive and invasive relapse rates are both 12.5%. 35% of early and locally advanced invasive breast cancers progress to advanced disease [41]. The recurrence rates for early and locally advanced breast cancer are 15.9% for node-positive [49] and 11% for node-negative disease [50]. Weighted for 31% node positive and 69% node negative, the composite recurrence rate for early and locally advanced breast cancer is 12.5%. The recurrence rate for the advanced disease is 66% (34% relapse-free five-year survival) [51].

Terminal care costs: The costs of terminal care for breast cancer are assumed to be the same as the costs of terminal care for ovarian cancer. In line with NICE recommendations future healthcare costs not associated with breast cancer ware not considered [23].

4.8. Cost of CHD

We used the ratio of breast cancer treatment costs in China compared to treatment costs in the UK to impute the costs of excess CHD and fatal CHD in China based on the cost of CHD in the UK.

5. Examination of Productivity Loss

The retirement ages for females are 50–55 in China and the female labour force participation rates are 62.03%, obtained from the World Bank [52]. The hourly wage rate is 5 USD dollars (2016 value) [53] and converted to 2019 values based on CPI in China.

We categorised the productivity costs as three subcomponents: (1) temporary disability due to short-term work absences following diagnosis, (2) permanent disability due to reduced working hours following a return to work or workforce departure; and (3) premature mortality due to death before retirement [54], detailed below.

Table S4. Descriptive statistics for productivity loss in breast and ovarian cancer patients.

Variables	Breast Cancer	Ovarian Cancer
(1) Temporary disability		
Percentage of temporary disability cases	94.0%	98%[1]
Average time taken off work following diagnosis (weeks)	44.9	47.22[2]
(2) Permanent disability		
Percentage of permanent disability: reduced hours	26%	40%[3]
Reduced hours per week after returning to work (hours)	5.5	5.5
(3) Premature mortality (before retirement)		
Percentage of permanent disability: workforce departure	12.9%	30%[3]

[1] We assume 98% ovarian cancer patients have cancer-related short-term work absences after diagnosis. [2] We assume ovarian cancer patients experience four weeks for surgery, 24 weeks for chemotherapy, and 24 weeks for recurrence treatment with the recurrence rate of 80% [55]. [3] We assume the percentages of permanent disability for ovarian cancer are 40% for reduced working hours and 30% for workforce departure. Temporary disability was calculated as time absent from work multiplied by age-specific gross earnings.

The descriptive statistics for productivity loss in breast cancer patients are obtained from Hanly et al. 2012 [54].

We calculated productivity costs due to permanent disability by applying age-specific gross earnings to the reduction in working hours, or the number of working hours if permanent workforce departure, until retirement age. Regarding productivity loss from premature mortality, we assumed that without cancer, the productive capacity of an individual would continue from the age of diagnosis until age of retirement. We multiplied the projected years of life lost by the age-specific gross earnings for the remainder of the working life to generate monetary estimates.

6. Estimates for AGE of onset and Survival for Breast and Ovarian Cancers

Our analysis incorporates lifetime risks and long-term consequences providing a lifetime time-horizon. Female lifetables from the World Health Organisation [20] were used.

We assumed that the median age for undergoing RRM and RRSO in unaffected path var carriers was 37 and 40 years respectively.[5] The uptake rates of RRSO and RRM are obtained from established literature [5,7]. OC/BC outcomes were modelled using five-year survival data. No statistically significant overall long-term survival differences between germline and sporadic OC/BC have been reported [56–58]. Five-year survival rate for BC is 83.2% (95% CI: 82.1, 84.3) and for OC is 41.8% (95% CI: 39.8, 43.7) [59]. After five-years, we assumed the probability of death for all patients was same as the general-population.

The excess risk of CHD following premenopausal oophorectomy is incorporated in the analysis [17,60]. We incorporated the fact that contralateral BC is associated with a higher risk of dying from BC [61].

7. Quality-Adjusted Life YEARS (QALYs) and Utility Scores.

QALY is a measurement of health-outcomes in economic evaluations recommended by NICE. It equals time spent in the relevant health states multiplied by an appropriate utility-score. Utility-score is an indication of individual preferences for specific health-states where 1 = perfect health and 0 = death. Utility-score is an adjustment for quality-of-life and QALY adjusts changes in length-of-life by potential alterations in quality-of-life. The utility-scores for early, advanced, recurrent, remittent, and end-stage BC are 0.79, 0.69, 0.45, 0.81, and 0.16 respectively[34,62]. The utility-scores for early, advanced, recurrent, remittent, and end-stage OC are 0.81, 0.55, 0.50, 0.83, and 0.16 respectively [63]. In addition, women undergoing RRM or RRSO also experience negative health-effects[64,65]. We used utility-scores of 0.88 (SD = 0.22) for RRM, 0.95 (SD = 0.10) for RRSO, and 0.84 (SD = 0.02) for CHD to account for the disutility[66,67].

8. Tornado Diagram – One-Way Sensitivity Analysis

(a)

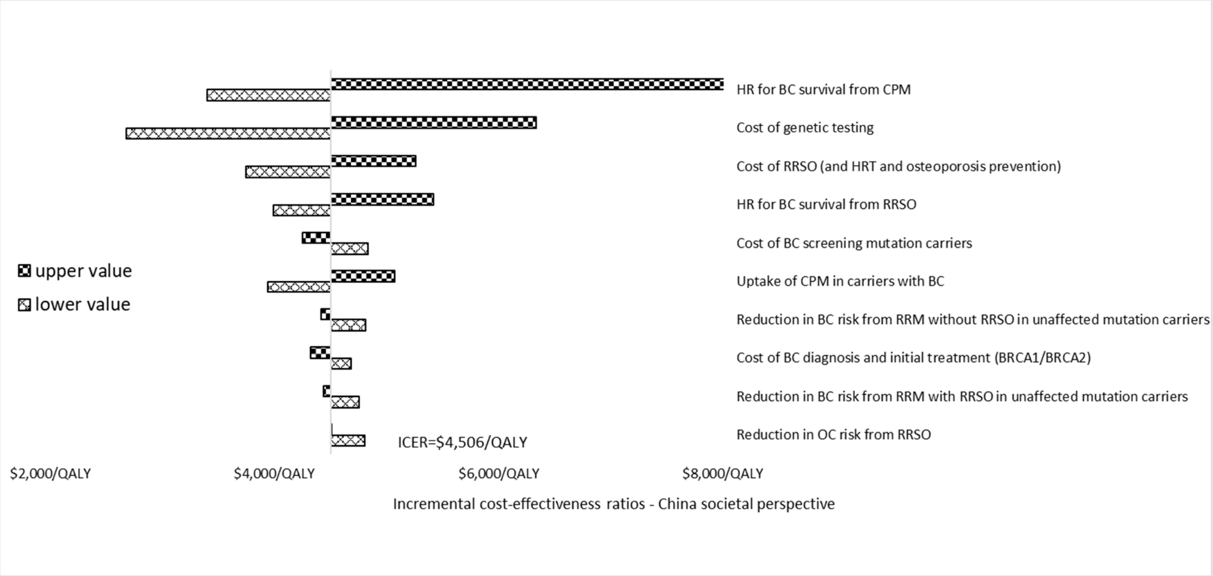

(b)

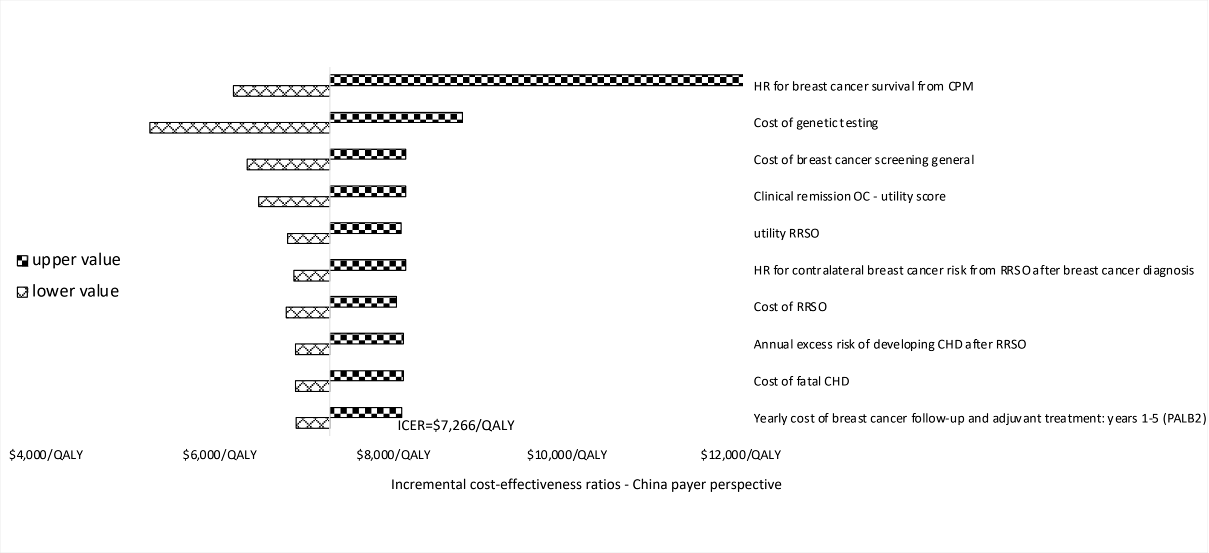

Figure S1. Tornado diagram – One-way Sensitivity Analysis. (**a**) One-way sensitivity analysis from societal perspective; (**b**) One-way sensitivity analysis from payer perspective.

BC – breast cancer, CPM – contralateral prophylactic mastectomy, HR – hazard ratio, ICER- incremental cost-effectiveness ration, OC – ovarian cancer, RRSO – risk-reducing salpingo-oophorectomy, RRM – risk-reducing mastectomy.

X-axis: Incremental cost-effectiveness ratio (ICER, $/QALY) (discounted).

Y-axis: Probability, cost and utility parameters in the model. The model is run at both lower and upper values/limits of the 95% confidence interval or range of all probability parameters described in Supplementary Table-1; and both lower and upper values/limits of the cost and utility-score parameters given in methods and Supplementary Table 3 and Table 6. Costs are varied by +/− 30%.

'Upper value' represents outcomes for upper limit and 'Lower value' represents outcomes for lower limit of the parameter.

References

1. Sun, J.; Meng, H.; Yao, L.; Lv, M.; Bai, J.; Zhang, J.; Wang, L.; Ouyang, T.; Li, J.; Wang, T.; et al. Germline Mutations in Cancer Susceptibility Genes in a Large Series of Unselected Breast Cancer Patients. *Clin. Cancer Res.* **2017**, *23*, 6113–6119, doi:10.1158/1078-0432.ccr-16-3227.
2. Slavin, T.P.; Maxwell, K.N.; Lilyquist, J.; Vijai, J.; Neuhausen, S.L.; Hart, S.N.; Ravichandran, V.; Thomas, T.; Maria, A.; Villano, D.; et al. The contribution of pathogenic variants in breast cancer susceptibility genes to familial breast cancer risk. *npj Breast Cancer* **2017**, *3*, 1–10, doi:10.1038/s41523-017-0024-8.
3. van Marcke, C.; Collard, A.; Vikkula, M.; Duhoux, F. Prevalence of pathogenic variants and variants of unknown significance in patients at high risk of breast cancer: A systematic review and meta-analysis of gene-panel data. *Crit. Rev. Oncol.* **2018**, *132*, 138–144, doi:10.1016/j.critrevonc.2018.09.009.
4. Mersch, J.; Brown, N.; Pirzadeh-Miller, S.; Mundt, E.; Cox, H.C.; Brown, K.; Aston, M.; Esterling, L.; Manley, S.; Ross, T. Prevalence of Variant Reclassification Following Hereditary Cancer Genetic Testing. *JAMA: J. Am. Med Assoc.* **2018**, *320*, 1266–1274, doi:10.1001/jama.2018.13152.
5. Evans, D.G.R.; Lalloo, F.; Ashcroft, L.; Shenton, A.; Clancy, T.; Baildam, A.D.; Brain, A.; Hopwood, P.; Howell, A. Uptake of Risk-Reducing Surgery in Unaffected Women at High Risk of Breast and Ovarian Cancer Is Risk, Age, and Time Dependent. *Cancer Epidemiology Biomarkers Prev.* **2009**, *18*, 2318–2324, doi:10.1158/1055-9965.epi-09-0171.
6. Evans, D.G.R.; Ingham, S.L.; Baildam, A.; Ross, G.L.; Lalloo, F.; Buchan, I.; Howell, A. Contralateral mastectomy improves survival in women with BRCA1/2-associated breast cancer. *Breast Cancer Res. Treat.* **2013**, *140*, 135–142, doi:10.1007/s10549-013-2583-1.
7. Manchanda, R.; Burnell, M.; Abdelraheim, A.; Johnson, M.; Sharma, A.; Benjamin, E.; Brunell, C.; Saridogan, E.; Gessler, S.; Oram, D.; et al. Factors influencing uptake and timing of risk reducing salpingo-oophorectomy in women at risk of familial ovarian cancer: a competing risk time to event analysis. *BJOG: Int. J. Obstet. Gynaecol.* **2012**, *119*, 527–536, doi:10.1111/j.1471-0528.2011.03257.x.
8. Kauff, N.D.; Domchek, S.M.; Friebel, T.M.; Robson, M.E.; Lee, J.; Garber, J.E.; Isaacs, C.; Evans, D.G.; Lynch, H.; Eeles, R.A.; et al. Risk-Reducing Salpingo-Oophorectomy for the Prevention of BRCA1- and BRCA2-Associated Breast and Gynecologic Cancer: A Multicenter, Prospective Study. *J. Clin. Oncol.* **2008**, *26*, 1331–1337, doi:10.1200/jco.2007.13.9626.
9. Rebbeck, T.R.; Friebel, T.; Lynch, H.T.; Neuhausen, S.L.; Veer, L.V. 'T; Garber, J.E.; Evans, G.; Narod, S.A.; Isaacs, C.; Matloff, E.; et al. Bilateral Prophylactic Mastectomy Reduces Breast Cancer Risk in BRCA1 and BRCA2 Mutation Carriers: The PROSE Study Group. *J. Clin. Oncol.* **2004**, *22*, 1055–1062, doi:10.1200/jco.2004.04.188.
10. Rebbeck, T.R.; Kauff, N.D.; Domchek, S.M. Meta-analysis of Risk Reduction Estimates Associated With Risk-Reducing Salpingo-oophorectomy in BRCA1 or BRCA2 Mutation Carriers. *J. Natl. Cancer Inst.* **2009**, *101*, 80–87, https://doi.org/10.1093/jnci/djn442.
11. Basu, N.N.; Ingham, S.; Hodson, J.; Lalloo, F.; Bulman, M.; Howell, A.; Evans, G. Risk of contralateral breast cancer in BRCA1 and BRCA2 mutation carriers: a 30-year semi-prospective analysis. *Fam. Cancer* **2015**, *14*, 531–538, doi:10.1007/s10689-015-9825-9.
12. Finch, A.; Beiner, M.; Lubinski, J.; Lynch, H.T.; Moller, P.; Rosen, B.; Murphy, J.; Ghadirian, P.; Friedman, E.; Foulkes, W.; et al. Salpingo-oophorectomy and the Risk of Ovarian, Fallopian Tube, and Peritoneal Cancers in Women With a BRCA1 or BRCA2 Mutation. *JAMA: J. Am. Med Assoc.* **2006**, *296*, 185–192, doi:10.1001/jama.296.2.185.
13. Metcalfe, K.; Lynch, H.T.; Foulkes, W.D.; Tung, N.; Kim-Sing, C.; Olopade, O.I.; Eisen, A.; Rosen, B.; Snyder, C.; Gershman, S.; et al. Effect of Oophorectomy on Survival After Breast Cancer inBRCA1andBRCA2Mutation Carriers. *JAMA Oncol.* **2015**, *1*, 306–313, doi:10.1001/jamaoncol.2015.0658.
14. Read, M.D.; A Edey, K.; Hapeshi, J.; Foy, C. Compliance with estrogen hormone replacement therapy after oophorectomy: a prospective study. *Menopause Int.* **2010**, *16*, 60–64, doi:10.1258/mi.2010.010023.
15. Cuzick, J.; Sestak, I.; Cawthorn, S.; Hamed, H.; Holli, K.; Howell, A.; Forbes, J.F.; IBIS-I Investigators. Tamoxifen for prevention of breast cancer: extended long-term follow-up of the IBIS-I breast cancer prevention trial. *Lancet Oncol.* **2015**, *16*, 67–75, https://doi.org/10.1016/s1470-2045(14)71171-4.
16. Smith, S.G.; Sestak, I.; Forster, A.; Partridge, A.; Side, L.; Wolf, M.S.; Horne, R.; Wardle, J.; Cuzick, J. Factors affecting uptake and adherence to breast cancer chemoprevention: a systematic review and meta-analysis. *Ann. Oncol.* **2016**, *27*, 575–590, doi:10.1093/annonc/mdv590.
17. Parker, W.H.; Feskanich, D.; Broder, M.S.; Chang, E.; Shoupe, D.; Farquhar, C.M.; Berek, J.S.; Manson, J.E. Long-Term Mortality Associated With Oophorectomy Compared With Ovarian Conservation in the Nurses' Health Study. *Obstet. Gynecol.* **2013**, *121*, 709–716, doi:10.1097/aog.0b013e3182864350.
18. United Nations Department of Economic and Social Affairs, World Population Prospects 2019. 2019. https://population.un.org/wpp/Download/Standard/Fertility/, (accessed on 07 Feb 2020).
19. International Agency for Research on Cancer. Estimated number of new cases in 2018, worldwide, females, all ages. 2018 [cited 2018 21 Nov]; Available from: http://gco.iarc.fr/today/online-analysis-table, (accessed on 07 Feb 2020).
20. World Health Organisation, Life tables. WHO: Geneva, Switzerland, 2016.
21. Chen, H.; Chen, Y.; Cui, B. The association of multimorbidity with healthcare expenditure among the elderly patients in Beijing, China. *Arch. Gerontol. Geriatr.* **2018**, *79*, 32–38, doi:10.1016/j.archger.2018.07.008.
22. The World Bank. PPP conversion factor, GDP. 2016. Available from: http://data.worldbank.org/indicator/PA.NUS.PPP, (accessed on 09 August 2020).

23. National Institute of Health and Clinical Excellence, Guide to the methods of technology appraisal. 2013: London. https://www.nice.org.uk/process/pmg9/resources/guide-to-the-methods-of-technology-appraisal-2013-pdf-2007975843781, (accessed on 15 June 2021).
24. Neuburger, J.; MacNeill, F.; Jeevan, R.; van der Meulen, J.H.P.; A Cromwell, D. Trends in the use of bilateral mastectomy in England from 2002 to 2011: retrospective analysis of hospital episode statistics. *BMJ Open* **2013**, *3*, e003179, doi:10.1136/bmjopen-2013-003179.
25. Del Corral, G.A.; Wes, A.M.; Fischer, J.P.; Serletti, J.M.; Wu, L.C. Outcomes and Cost Analysis in High-Risk Patients Undergoing Simultaneous Free Flap Breast Reconstruction and Gynecologic Procedures. *Ann. Plast. Surg.* **2015**, *75*, 534–538, doi:10.1097/sap.0000000000000156.
26. Miller, M.E.; Czechura, T.; Martz, B.; Hall, M.E.; Pesce, C.; Jaskowiak, N.; Winchester, D.J.; Yao, K. Operative Risks Associated with Contralateral Prophylactic Mastectomy: A Single Institution Experience. *Ann. Surg. Oncol.* **2013**, *20*, 4113–4120, doi:10.1245/s10434-013-3108-1.
27. Breast cancer screening guideline for Chinese women. *Cancer Biol. Med.* **2019**, *16*, 822–824, doi:10.20892/j.issn.2095-3941.2019.0321.
28. National Institute for Health and Care Excellence, Familial breast cancer: Classification and care of people at risk of familial breast cancer and management of breast cancer and related risks in people with a family history of breast cancer, in NICE clinical guideline CG164 ed. 2013, National Institute for Health and Care Excellence: London, UK. https://www.nice.org.uk/guidance/cg164, (accessed on 08 Aug 2017).
29. Cuzick, J.; Sestak, I.; Bonanni, B.; Costantino, J.P.; Cummings, S.; DeCensi, A.; Dowsett, M.; Forbes, J.F.; Ford, L.; LaCroix, A.Z.; et al. Selective oestrogen receptor modulators in prevention of breast cancer: An updated meta-analysis of individual participant data. *Lancet* **2013**, *381*, 1827–1834, doi:10.1016/s0140-6736(13)60140-3.
30. Wang, Q.; Li, J.; Zheng, S.; Li, J.-Y.; Pang, Y.; Huang, R.; Zhang, B.-N.; Zhang, B.; Yang, H.-J.; Xie, X.-M.; et al. Breast cancer stage at diagnosis and area-based socioeconomic status: a multicenter 10-year retrospective clinical epidemiological study in China. *BMC Cancer* **2012**, *12*, 122, doi:10.1186/1471-2407-12-122.
31. Heijnsdijk, E.A.; Warner, E.; Gilbert, F.; Tilanus-Linthorst, M.M.; Evans, G.; Causer, P.A.; Eeles, R.; Kaas, R.; Draisma, G.; Ramsay, E.A.; et al. Differences in Natural History between Breast Cancers in BRCA1 and BRCA2 Mutation Carriers and Effects of MRI Screening-MRISC, MARIBS, and Canadian Studies Combined. *Cancer Epidemiology Biomarkers Prev.* **2012**, *21*, 1458–1468, doi:10.1158/1055-9965.epi-11-1196.
32. Nelson, H.D.; Pappas, M.; Zakher, B.; Mitchell, J.P.; Okinaka-Hu, L.; Fu, R. Risk Assessment, Genetic Counseling, and Genetic Testing for BRCA-Related Cancer in Women: A Systematic Review to Update the U.S. Preventive Services Task Force Recommendation. *Ann. Intern. Med.* **2014**, *160*, 255–266, doi:10.7326/m13-1684.
33. National Institute for Health and Care Excellence (NICE), Early and locally advanced breast cancer: diagnosis and treatment. 2009, National Institute for Health and Clinical Excellence: Cardiff, Wales, UK. https://www.nice.org.uk/guidance/CG80/evidence, (accessed on 15 June 2018).
34. National Institute for Health and Care Excellence (NICE), Advanced breast cancer: diagnosis and treatment. 2009, National Institute for Health and Clinical Excellence: Cardiff, Wales, UK. https://www.nice.org.uk/guidance/cg81, (accessed on 15 June 2018).
35. Cortesi, L.; Turchetti, D.; Marchi, I.; Fracca, A.; Canossi, B.; Battista, R.; Ruscelli, S.; Pecchi, A.R.; Torricelli, P.; Federico, M. Breast cancer screening in women at increased risk according to different family histories: an update of the Modena Study Group experience. *BMC Cancer* **2006**, *6*, 210, doi:10.1186/1471-2407-6-210.
36. MARIBS Study Group. Screening with magnetic resonance imaging and mammography of a UK population at high familial risk of breast cancer: a prospective multicentre cohort study (MARIBS). *Lancet* **2005**, *365*, 1769–1778, https://doi.org/10.1016/s0140-6736(05)66481-1.
37. Robson, M.; O Chappuis, P.; Satagopan, J.; Wong, N.; Boyd, J.; Goffin, J.R.; Hudis, C.; Roberge, D.; Norton, L.; Bégin, L.R.; et al. A combined analysis of outcome following breast cancer: differences in survival based on BRCA1/BRCA2 mutation status and administration of adjuvant treatment. *Breast Cancer Res.* **2003**, *6*, 1–10, doi:10.1186/bcr658.
38. Comen, E.; Davids, M.; Kirchhoff, T.; Hudis, C.; Offit, K.; Robson, M. Relative contributions of BRCA1 and BRCA2 mutations to “triple-negative” breast cancer in Ashkenazi Women. *Breast Cancer Res. Treat.* **2011**, *129*, 185–190, doi:10.1007/s10549-011-1433-2.
39. Tung, N.; Garber, J.E.; Lincoln, A.; Domchek, S.M. Frequency of Triple-Negative Breast Cancer inBRCA1Mutation Carriers: Comparison Between Common Ashkenazi Jewish and Other Mutations. *J. Clin. Oncol.* **2012**, *30*, 4447–4448, doi:10.1200/jco.2012.44.5635.
40. Chappuis, P.O.; Nethercot, V.; Foulkes, W.D. Foulkes, Clinico-pathological characteristics of BRCA1- and BRCA2-related breast cancer. *Semin. Surg. Oncol.*, **2000**, *18*, 287–295.
41. National Institute for Health and Clinical Excellence, National costing report: Early and locally advanced breast cancer/Advanced breast cancer. 2009, National Institute for Health and Clinical Excellence: London, UK. Website Link, (accessed on).
42. Bates, T.; Kearins, O.; Monypenny, I.; Lagord, C.; Lawrence, G. Clinical outcome data for symptomatic breast cancer: the breast cancer clinical outcome measures (BCCOM) project. *Br. J. Cancer* **2009**, *101*, 395–402, doi:10.1038/sj.bjc.6605155.

43. Early Breast Cancer Trialists' Collaborative Group (EBCTCG) Effects of chemotherapy and hormonal therapy for early breast cancer on recurrence and 15-year survival: an overview of the randomised trials. *Lancet* **2005**, *365*, 1687–1717, https://doi.org/10.1016/s0140-6736(05)66544-0.
44. Jeevan, R.; Mennie, J.C.; Mohanna, P.N.; O'Donoghue, J.M.; Rainsbury, R.M.; Cromwell, D.A. National trends and regional variation in immediate breast reconstruction rates. *Br. J. Surg.* **2016**, *103*, 1147–1156, doi:10.1002/bjs.10161.
45. Blamey, R.; Ellis, I.; Pinder, S.; Lee, A.; Macmillan, R.; Morgan, D.; Robertson, J.; Mitchell, M.; Ball, G.; Haybittle, J.; et al. Survival of invasive breast cancer according to the Nottingham Prognostic Index in cases diagnosed in 1990–1999. *Eur. J. Cancer* **2007**, *43*, 1548–1555, doi:10.1016/j.ejca.2007.01.016.
46. Jonathan, G. and D. Robyn, Adjuvant! Online: review of evidence concerning its validity, and other considerations relating to its use in the NHS, in NICE Clinical Guidelines, No.80: Early and locally advanced breast cancer: diagnosis and treatment. 2009, National Institute for Health and Clinical Excellence: Cardiff, Wales, UK. Website Link, (accessed on).
47. Kozlow, W.; Guise, T.A. Breast Cancer Metastasis to Bone: Mechanisms of Osteolysis and Implications for Therapy. *J. Mammary Gland Biol. Neoplasia* **2005**, *10*, 169–180, doi:10.1007/s10911-005-5399-8.
48. Cancer Research UK, Breast Cancer (C50), One-Year Age Standardised Net Survival by Stage, Adults (Ages 15–99 Years), England 2014. 2014. https://www.cancerresearchuk.org/health-professional/cancer-statistics/statistics-by-cancer-type/breast-cancer/survival, (accessed on 15 June 2018).
49. Wapnir, I.L.; Anderson, S.; Mamounas, E.P.; Jr, C.E.G.; Jeong, J.; Tan-Chiu, E.; Fisher, B.; Wolmark, N. Prognosis After Ipsilateral Breast Tumor Recurrence and Locoregional Recurrences in Five National Surgical Adjuvant Breast and Bowel Project Node-Positive Adjuvant Breast Cancer Trials. *J. Clin. Oncol.* **2006**, *24*, 2028–2037, doi:10.1200/jco.2005.04.3273.
50. Anderson, S.J.; Wapnir, I.; Dignam, J.J.; Fisher, B.; Mamounas, E.P.; Jeong, J.; Jr, C.E.G.; Wickerham, D.L.; Costantino, J.P.; Wolmark, N. Prognosis After Ipsilateral Breast Tumor Recurrence and Locoregional Recurrences in Patients Treated by Breast-Conserving Therapy in Five National Surgical Adjuvant Breast and Bowel Project Protocols of Node-Negative Breast Cancer. *J. Clin. Oncol.* **2009**, *27*, 2466–2473, doi:10.1200/jco.2008.19.8424.
51. Gennari, A.; Conte, P.; Rosso, R.; Orlandini, C.; Bruzzi, P. Survival of metastatic breast carcinoma patients over a 20-year period. *Cancer* **2005**, *104*, 1742–1750, doi:10.1002/cncr.21359.
52. The World Bank. Labor force participation rate, female (% of female population ages 15+) (modeled ILO estimate). 2018; Available from: https://data.worldbank.org/indicator/SL.TLF.CACT.FE.ZS. (accessed on 07 Nov 2018).
53. World Economic Forum. The Global Gender Gap Report 2016. 2016; Available from: http://reports.weforum.org/global-gender-gap-report-2016/economies/#economy=CHN. (accessed on 07 Nov 2018).
54. Hanly, P.; Timmons, A.; Walsh, P.M.; Sharp, L. Breast and Prostate Cancer Productivity Costs: A Comparison of the Human Capital Approach and the Friction Cost Approach. *Value Heal.* **2012**, *15*, 429–436, doi:10.1016/j.jval.2011.12.012.
55. National Ovarian Cancer Coalition. Ovarian Cancer Recurrence: Discussion With an Expert. 2016; Available from: http://ovarian.org/component/content/article/33/385. (accessed on 07 Nov 2018).
56. Bordeleau, L.; Panchal, S.; Goodwin, P. Prognosis of BRCA-associated breast cancer: a summary of evidence. *Breast Cancer Res. Treat.* **2009**, *119*, 13–24, doi:10.1007/s10549-009-0566-z.
57. Rennert, G.; Bisland-Naggan, S.; Barnett-Griness, O.; Bar-Joseph, N.; Zhang, S.; Rennert, H.S.; Narod, S.A. Clinical Outcomes of Breast Cancer in Carriers ofBRCA1andBRCA2Mutations. *New Engl. J. Med.* **2007**, *357*, 115–123, doi:10.1056/nejmoa070608.
58. McLaughlin, J.R.; Rosen, B.; Moody, J.; Pal, T.; Fan, I.; Shaw, P.A.; Risch, H.A.; Sellers, T.A.; Sun, P.; Narod, S.A. Long-Term Ovarian Cancer Survival Associated With Mutation in BRCA1 or BRCA2. *JNCI: J. Natl. Cancer Inst.* **2013**, *105*, 141–148, doi:10.1093/jnci/djs494.
59. Allemani, C.; Matsuda, T.; Di Carlo, V.; Harewood, R.; Matz, M.; Nikšić, M.; Bonaventure, A.; Valkov, M.; Johnson, C.J.; Estève, J.; et al. Global surveillance of trends in cancer survival 2000–14 (CONCORD-3): analysis of individual records for 37 513 025 patients diagnosed with one of 18 cancers from 322 population-based registries in 71 countries. *Lancet* **2018**, *391*, 1023–1075, https://doi.org/10.1016/s0140-6736(17)33326-3.
60. Parrish, H.M.; Carr, C.A.; Hall, D.G.; King, T.M. Time interval from castration in premenopausal women to development of excessive coronary atherosclerosis. *Am. J. Obstet. Gynecol.* **1967**, *99*, 155–162, doi:10.1016/0002-9378(67)90314-6.
61. Vichapat, V.; Garmo, H.; Holmqvist, M.; Liljegren, G.; Wärnberg, F.; Lambe, M.; Fornander, T.; Adolfsson, J.; Lüchtenborg, M.; Holmberg, L. Tumor Stage Affects Risk and Prognosis of Contralateral Breast Cancer: Results From a Large Swedish-Population–Based Study. *J. Clin. Oncol.* **2012**, *30*, 3478–3485, doi:10.1200/jco.2011.39.3645.
62. Shi, J.-F.; Huang, H.-Y.; Guo, L.-W.; Shi, D.; Gu, X.-Y.; Liang, H.; Wang, L.; Ren, J.-S.; Bai, Y.-N.; Mao, A.-Y.; et al. Quality-of-life and health utility scores for common cancers in China: a multicentre cross-sectional survey. *Lancet* **2016**, *388*, S29, doi:10.1016/s0140-6736(16)31956-0.
63. Havrilesky, L.J.; Broadwater, G.; Davis, D.M.; Nolte, K.C.; Barnett, J.C.; Myers, E.R.; Kulasingam, S. Determination of quality of life-related utilities for health states relevant to ovarian cancer diagnosis and treatment. *Gynecol. Oncol.* **2009**, *113*, 216–220, doi:10.1016/j.ygyno.2008.12.026.
64. Grann, V.R.; Patel, P.; Bharthuar, A.; Jacobson, J.S.; Warner, E.; Anderson, K.; Warner, E.; Tsai, W.-Y.; Hill, K.A.; Neugut, A.I.; et al. Breast cancer-related preferences among women with and without BRCA mutations. *Breast Cancer Res. Treat.* **2009**, *119*, 177–184, doi:10.1007/s10549-009-0373-6.
65. Parker, W.H.; Jacoby, V.; Shoupe, D.; Rocca, W. Effect of Bilateral Oophorectomy on Women's Long-Term Health. *Women's Heal.* **2009**, *5*, 565–576, doi:10.2217/whe.09.42.

66. Grann, V.R.; Patel, P.R.; Jacobson, J.S.; Warner, E.; Heitjan, D.F.; Ashby-Thompson, M.; Hershman, D.L.; Neugut, A.I. Comparative effectiveness of screening and prevention strategies among BRCA1/2-affected mutation carriers. *Breast Cancer Res. Treat.* **2010**, *125*, 837–847, doi:10.1007/s10549-010-1043-4.
67. van Kempen, B.J.; Spronk, S.; Koller, M.T.; Elias-Smale, S.E.; Fleischmann, K.E.; Ikram, M.A.; Krestin, G.P.; Hofman, A.; Witteman, J.C.; Hunink, M.M. Comparative Effectiveness and Cost-Effectiveness of Computed Tomography Screening for Coronary Artery Calcium in Asymptomatic Individuals. *J. Am. Coll. Cardiol.* **2011**, *58*, 1690–1701, doi:10.1016/j.jacc.2011.05.056.
